# Skin immune microenvironment in psoriasis: from bench to bedside

**DOI:** 10.3389/fimmu.2025.1643418

**Published:** 2025-08-29

**Authors:** Yi Yao, Li-Qing Chen, Yi-Bo Lv, Shun-Li Tang, Wei Shen, Hui Sun, Hua-Jie Zhong

**Affiliations:** ^1^ Department of Dermatology, Huzhou Central Hospital, Affiliated Central Hospital of Huzhou University, Huzhou, Zhejiang, China; ^2^ Department of Dermatology, Huzhou Central Hospital, Fifth School of Clinical Medicine of Zhejiang Chinese Medical University, Huzhou, Zhejiang, China

**Keywords:** psoriasis, immune microenvironment, pathogenesis, cytokines, biologics, translational research, gut microbiota, neuroimmunology

## Abstract

Psoriasis, a chronic immune-mediated inflammatory skin disorder affecting approximately 2-3% of the global population, manifests in distinct forms including plaque, pustular, and erythrodermic types. The pathogenesis involves complex interactions between genetic susceptibility, epigenetic modifications, and environmental triggers that disrupt immune homeostasis, particularly within the skin’s epithelial immune microenvironment (EIME). This review examines the fundamental mechanisms of psoriasis from a ‘bench’ perspective, encompassing genetic triggers, immune cell contributions, cytokine cascades, and insights derived from multi-omics studies. It also incorporates emerging areas such as gut microbiota dysbiosis and neuro-immunological influences. Translational research linking these discoveries to clinical application is discussed, covering biomarker identification, comorbidity management, and the advancement of novel therapies. At the ‘bedside’, we evaluate current conventional treatments, targeted biologic agents (e.g., TNF-α, IL-17, and IL-23 inhibitors), and emerging modalities including JAK inhibitors, epigenetic modulators, and stem cell therapies. Challenges pertaining to efficacy, safety, and personalized medicine are addressed, alongside future directions emphasizing multi-omics integration and holistic immune targeting. Highlighting the critical role of the immune microenvironment, this narrative review underscores the translational progress driving towards improved patient outcomes.

## 1 Introduction

Psoriasis is a chronic, immune-mediated dermatosis affecting approximately 125 million people worldwide and is characterized by well-demarcated erythematous plaques surmounted by silvery-white scales (1, 2). Disease initiation and perpetuation arise from a complex interplay between polygenic risk variants—notably HLA-C*06:02 and PSORS susceptibility loci—and dynamic epigenetic reprogramming triggered by environmental insults such as β-hemolytic streptococcal infection, psychological stress, and visceral adiposity (3, 4). Within the epidermal immune microenvironment, pathogenic circuits converge on the IL-23/IL-17 axis. Activated dermal dendritic cells secrete IL-23 that licenses CD8^+^ Tc17 and γδ T cells to produce IL-17A and IL-22, while neutrophil extracellular traps amplify keratinocyte-derived IL-36 and the chemokine CCL20, thereby sustaining a self-propagating inflammatory loop (5–7). Macrophages, natural killer cells, and B cells further contribute cytokines and autoantibodies that reinforce tissue remodeling and barrier dysfunction (7). This persistent inflammatory tone extends beyond skin, engaging systemic pathways—including neuro-immune crosstalk—that underlie recognized comorbidities such as depression and cardiovascular disease (8–11). Current therapeutic algorithms encompass topical corticosteroids and vitamin D analogs, narrow-band UVB phototherapy, conventional immunosuppressants (methotrexate, cyclosporine), and increasingly, targeted biologics and small molecules that inhibit TNF-α, IL-17A/F, IL-23p19, or intracellular kinases (e.g., TYK2) (12–15). Despite substantial advances, heterogeneous response rates, cumulative toxicities, secondary loss of efficacy, and high economic burden remain important unmet needs. This review employs a “bench-to-bedside” approach to synthesize immunological insights with clinical progress, with the goal of optimizing therapeutic strategies for psoriasis.

## 2 Pathogenesis of psoriasis: basic mechanisms

### 2.1 Genetic and environmental triggers

Psoriasis is underpinned by a robust genetic framework, with genome-wide association studies (GWAS) identifying over 80 susceptibility loci that drive immune dysregulation and disease predisposition. Key among these is HLA-Cw6, strongly associated with early-onset psoriasis within the PSORS1 region on chromosome 6p21, alongside other PSORS loci and genes regulating cytokine signaling, such as *IL12B* and *IL23R*, as well as NF-κB pathway components like *TNFAIP3* and *NFKBIA*, which orchestrate inflammatory responses and epidermal proliferation (3, 16, 17). These genetic variants synergistically heighten susceptibility, particularly in individuals with a familial history, where heritability estimates approach 80% (18). Epigenetic mechanisms amplify this genetic predisposition by modulating gene expression without altering the DNA sequence. Aberrant DNA methylation patterns in immune-related genes, histone modifications that alter chromatin accessibility, and dysregulated non-coding RNAs, including long non-coding RNAs (lncRNAs) such as MEG3, significantly influence keratinocyte differentiation and immune cell activation in psoriatic lesions (19–21). These epigenetic alterations, which may be inherited or induced by environmental factors, perpetuate chronic inflammation.

Environmental triggers are critical in unmasking genetic and epigenetic vulnerabilities. Streptococcal infections, for instance, precipitate guttate psoriasis through molecular mimicry, while obesity, smoking, excessive alcohol consumption, and psychological stress activate innate immune pathways, leading to elevated cytokine production, oxidative stress, and recurrent disease flares (22–24). These factors disrupt epidermal homeostasis and initiate inflammatory cascades within the skin immune microenvironment, exacerbating psoriatic pathology.

### 2.2 The skin immune microenvironment

**Figure 1:**
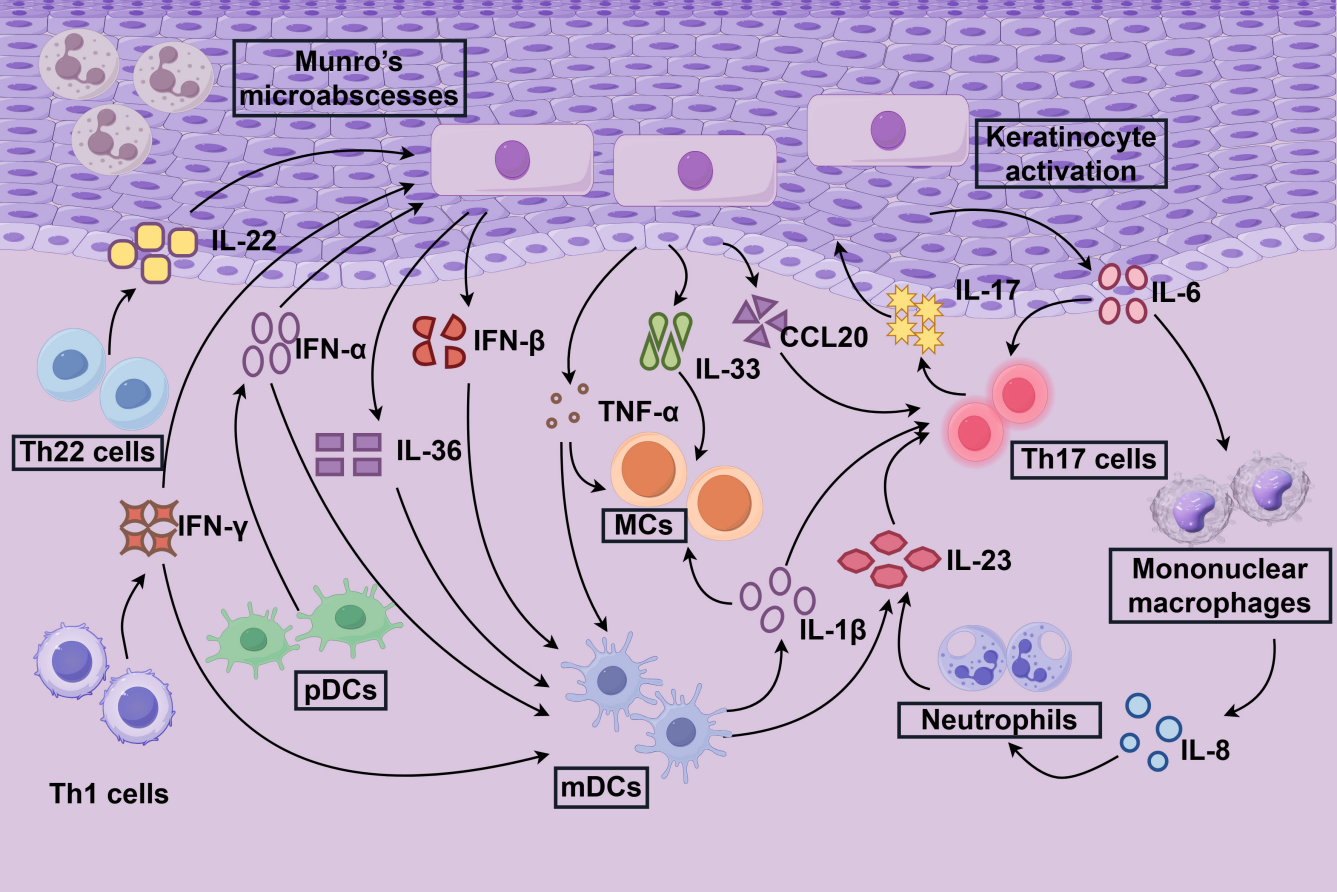
Immune response mechanisms in psoriasis. The immune response within the localized inflammatory microenvironment of psoriasis, termed the “inflammazone” is illustrated. Keratinocytes play a pivotal role in initiating psoriasis by acquiring immune cell-like functions upon activation. Stimulated by cytokines such as IL-17, IL-22, and IFN-γ, keratinocytes trigger a cascade of inflammatory responses in the skin. This process recruits mononuclear macrophages, neutrophils, and dendritic cells (DCs), which amplify inflammation through the secretion of cytokines, including IL-13, IL-23, and TNF-α. Central to this inflammatory cascade are Th17, Th22, and Th1 cells, whose activation and persistence are driven by cytokines such as IL-23, perpetuating the chronic inflammatory state characteristic of psoriasis.

The skin immune microenvironment is a dynamic and finely tuned ecosystem encompassing the epidermis and dermis. The epidermis primarily comprises keratinocytes and Langerhans cells, with the latter functioning as key antigen-presenting cells. In contrast, the dermis harbors a diverse array of immune cells, including dendritic cells, macrophages, and sensory nerves that facilitate neuro-immune interactions and cytokine signaling (25, 26). Under normal physiological conditions, this intricate network maintains immune homeostasis through vigilant immune surveillance and tolerance mechanisms, effectively preventing unwarranted inflammation. In psoriasis, the pathogenesis is multifaceted, with the cutaneous immune microenvironment playing a pivotal role. This environment is intricately linked to the aberrant activation of various immune cells, driving a complex inflammatory cascade that perpetuates the disease (5, 6) (**Figure 1**).

In psoriasis, the delicate balance of the skin immune microenvironment is profoundly disrupted, leading to aberrant keratinocyte proliferation and differentiation. Under normal conditions, keratinocytes arise from stem cells in the basal layer of the epidermis, progressively maturing through the spinous and granular layers to form the stratum corneum. In psoriatic lesions, however, keratinocytes exhibit hyperproliferation and accelerated differentiation, resulting in epidermal thickening (acanthosis) and the formation of characteristic silvery scales (1). This dysregulation is driven by an inflammatory microenvironment in which keratinocytes secrete a plethora of pro-inflammatory mediators, including IL-1α, IL-6, IL-8, TNF-α, IL-17F, IL-23, and calcitonin gene-related peptide (CGRP). These mediators sustain inflammation through autocrine and paracrine signaling loops (5). Additionally, elevated reactive oxygen species (ROS) induce oxidative stress and DNA damage, further amplifying inflammation and cellular hyperproliferation. Compromised intercellular junctions, arising from dysregulated adhesion molecule expression, weaken the epidermal barrier, facilitating the infiltration of inflammatory cells (2).

Clinically, psoriasis is characterized by keratinocyte hyperproliferation, epidermal acanthosis, aberrant neovascularization, and chronic inflammation perpetuated by immune cell infiltrates (8, 12). Activated keratinocytes exacerbate this cascade by releasing chemokines, such as CCL20 and CXCL1, which recruit T cells and neutrophils, and cytokines, including IL-36 and IL-1β, which establish self-sustaining feedback loops with immune cells (6).

Emerging technologies, including single-cell and spatial transcriptomics, have illuminated these interactions. These approaches reveal spatially organized clusters of fibroblasts, keratinocytes, and immune cells that maintain a pro-inflammatory niche. This milieu features disrupted extracellular matrix remodeling and increased vascular permeability, collectively perpetuating the chronic inflammatory hallmark of psoriasis (27).

### 2.3 Key immune cells and their roles

#### 2.3.1 Innate immune cells

Innate immune cells orchestrate the initial response in psoriasis by detecting danger signals and amplifying inflammation to engage adaptive immune components. This diverse cellular repertoire includes dendritic cells (DCs), macrophages, neutrophils, natural killer (NK) cells, mast cells, innate lymphoid cells (ILCs), and myeloid-derived suppressor cells (MDSCs), which collectively drive pro-inflammatory cytokine production and tissue remodeling. As frontline defenders, these cells initiate early inflammatory responses that bridge to adaptive immunity. Dendritic cells, encompassing subsets such as slanDCs and plasmacytoid DCs, sense microbial or self-antigens through Toll-like receptors (TLRs), triggering the release of cytokines like IL-23, IL-12, and IFN-α, which activate T cells. Their increased presence in psoriatic lesions correlates closely with disease severity and plaque formation (28–30). Macrophages, polarized toward the pro-inflammatory M1 phenotype, secrete TNF-α, IL-1β, IL-6, and IL-23, promoting Th17 cell differentiation and intensifying dermal inflammation (31, 32). Neutrophils, abundant in psoriatic lesions, form neutrophil extracellular traps (NETs) and release IL-17, exacerbating acute symptoms, particularly in pustular psoriasis variants (33). Myeloid-derived suppressor cells (MDSCs) accumulate within the inflammatory microenvironment, producing IL-23 and IL-6 to skew the Th17/Treg balance toward a pro-inflammatory state (34). Mast cells, through a process termed MCETosis, release IL-17 and IL-33, while innate lymphoid cells (ILCs), particularly ILC3 subsets, contribute IL-17 and IL-22 during early inflammatory flares, linking innate responses to tissue remodeling (35, 36).

##### 2.3.1.1 Dendritic cells

Dendritic cells, pivotal antigen-presenting cells, orchestrate the pathogenesis and therapeutic modulation of psoriasis by initiating adaptive immune inflammatory responses (5). Originating primarily from bone marrow hematopoietic precursor cells, DCs migrate to the skin and differentiate into distinct subpopulations, including Langerhans cells (LCs) in the epidermis and inflammatory DCs in the dermis. Research demonstrates a significant enrichment of DCs in psoriatic lesions, where they activate Th17 cells via IL-23, triggering the release of pro-inflammatory cytokines such as IL-17A and establishing a pathological immune cycle (6). DC subpopulations exhibit functional heterogeneity in psoriasis (29, 37). Epidermal-resident Langerhans cells display impaired migratory capacity in non-lesional skin of patients (38), potentially contributing to early inflammatory initiation through aberrant antigen presentation and intercellular interactions (25). Semi-mature DCs, characterized by elevated IL1B expression, form a pro-inflammatory network with T cells through IL-1β ligand-receptor interactions (37, 39). Plasmacytoid dendritic cells (pDCs) amplify inflammation via the IL-36 signaling pathway and type I interferon responses (40, 41), particularly during acute disease flares. Notably, the LL37-DNA/RNA complex released by neutrophils specifically activates pDCs (30), perpetuating a self-sustaining inflammatory loop.

##### 2.3.1.2 Neutrophils

Neutrophils are pivotal in the pathogenesis of psoriasis, primarily originating from the peripheral blood circulatory system (42). In patients with psoriasis, CD10+ neutrophil subpopulations are markedly elevated in peripheral blood, including a subset resembling senescent neutrophils, which are approximately three times more abundant than in healthy individuals (42). These neutrophils exhibit distinct phenotypic characteristics and functional heterogeneity. Notably, the CXCR4+ neutrophil subpopulation is significantly increased in both the blood and inflamed skin of patients, correlating positively with disease severity (43). Upon activation, neutrophils propel the autoinflammatory cycle by releasing granular stores of IL-26, particularly prominent in pustular psoriasis. IL-26 stimulates keratinocytes to express IL-1 family cytokines and chemokines, further recruiting neutrophils to infiltrate the lesions (44). Neutrophils also contribute to pathogenesis by forming neutrophil extracellular traps (NETs), which release LL37-DNA complexes that activate the TLR9 pathway (30, 45). NET formation is regulated by the SHP2-ERK5 signaling pathway, driving local infiltration of pro-inflammatory cytokines (e.g., TNF-α, IL-1β, IL-6, IL-17A, and CXCL15) and exacerbating the vicious cycle between keratinocytes and neutrophils (46). Neutrophil-derived IL-1β plays a central role in the psoriasis model, promoting IL-17A production through an estrogen receptor-dependent mechanism (47). Locally, the co-localization of neutrophils with T cells in the skin induces IL-17 and IFN-γ production by T cells via NETs (42). Additionally, the enhanced glycolytic metabolism and lactate-releasing properties of CXCR4+ neutrophils promote vascular permeability and tissue remodeling (43). Biologic therapies effectively reduce the number of activated neutrophils in circulation, underscoring their critical role in sustaining persistent inflammation (42).

##### 2.3.1.3 Macrophages

In psoriasis pathogenesis, macrophages, primarily derived from bone marrow monocytes, migrate via the circulatory system to sites of cutaneous inflammation. Within psoriatic lesions, these cells are activated by local microenvironmental cues, such as IL-23, adopting a distinct polarization state that diverges from both classical pro-inflammatory M1 and anti-inflammatory M2 phenotypes (32). IL-23-stimulated macrophages exhibit unique gene expression profiles and secrete elevated levels of pro-inflammatory mediators, including IFN-γ, which significantly drive dermatitis progression in psoriasis-like mouse models (32). During inflammation, activated macrophages release key cytokines, such as TNF and IL-12p40. Notably, increased expression of MCPIP3 in macrophages enhances TNF and IL-12p40 production, directly exacerbating cutaneous inflammatory responses (48). Concurrently, macrophage efferocytosis in psoriatic lesions is markedly impaired, hindering the clearance of apoptotic cells. This dysfunction establishes a vicious cycle: impaired efferocytosis activates platelets, which, in turn, suppress macrophage phagocytic receptor expression, further amplifying inflammation (49). Macrophages also engage in close interactions with keratinocytes, promoting their hyperproliferation through mediators like TNF and cytokines such as IL-25/IL-17E. These factors further activate macrophages and other immune cells, perpetuating a positive feedback loop of inflammatory signaling (5, 50). Moreover, macrophage polarization can be modulated via the TGR5 receptor pathway. For instance, the TGR5 agonist sauchinone inhibits M1 polarization and mitigates imiquimod (IMQ)-induced psoriasis-like dermatitis. This protective effect is abolished in Tgr5 knockout mice, underscoring the critical role of TGR5-mediated regulation of macrophage function in modulating disease progression (51).

##### 2.3.1.4 Natural killer cells

Natural killer cells, as key innate immune lymphocytes, play a critical role in modulating inflammation within the psoriatic skin microenvironment. Originating primarily from bone marrow hematopoietic precursor cells, NK cells in psoriatic lesions coexist with T-cell subpopulations, dendritic cells, melanocytes, and keratinocytes across various skin layers, as revealed by unsupervised clustering analyses. Their abundance is significantly elevated in lesional skin compared to non-lesional and healthy skin (6, 27). In the immunopathology of psoriasis, NK cells contribute to a dysregulated interplay between innate and adaptive immunity. They form a complex network with other immune cells, secreting pro-inflammatory cytokines and exerting direct cytotoxic effects that amplify local inflammatory responses (52, 53). However, in the chronic inflammatory milieu of psoriasis, NK cell function is often suppressed. An imbalance in surface receptor expression, such as the upregulation of inhibitory receptors like KLRG1, diminishes their capacity to eliminate aberrant keratinocytes (54). Additionally, IL-25 (IL-17E) secreted by keratinocytes further activates NK cells and other immune cells, perpetuating an inflammatory cycle (55). Intraepidermal NK cells also engage in physical interactions with Langerhans cells (LCs), collaboratively orchestrating T cell-mediated inflammatory responses and exacerbating epidermal hyperplasia (25). Collectively, NK cells in psoriasis drive a chronic inflammatory cascade centered on IL-17 through cytokine secretion, intercellular interactions, and functional impairments.

#### 2.3.2 Adaptive immune cells

Adaptive immune cells drive the chronicity of psoriasis through antigen-specific responses and immune memory, encompassing T cell subsets (CD4+, CD8+, γδ T, Th1, Th17, Th22, Th9, Treg, and tissue-resident memory T cells [TRM]), B cells, and regulatory mechanisms. These cells perpetuate inflammation through targeted cytokine release and sustained effector functions. CD4+ T cells are predominant, with Th1 cells producing IFN-γ to bolster antimicrobial responses, Th17 cells secreting IL-17A/F as central pathogenic mediators, Th22 cells releasing IL-22 to promote epidermal hyperplasia, and Th9 cells contributing IL-9 to amplify inflammation (5, 6, 56). Concurrently, regulatory T cell (Treg) dysfunction, driven by IL-6, leads to their conversion into pro-inflammatory effectors, further exacerbating the inflammatory milieu (6, 56).CD8+ T cells, particularly Tc17 and TRM subsets, infiltrate the epidermis, releasing IL-17 and IFN-γ to sustain local inflammation and facilitate disease recurrence upon environmental triggers (57, 58). γδ T cells bridge innate and adaptive immunity, rapidly producing IL-17, IL-22, and chemokines during early disease stages to recruit effector cells and modulate keratinocyte responses (59, 60). Although less extensively studied, B cells are increased in psoriatic lesions and may contribute to humoral pathology by producing autoantibodies or supporting T cell activation, despite their primarily supportive role (61).

##### 2.3.2.1 Th1 and Th17 cells

In psoriasis, Th1 and Th17 cells, as pivotal CD4+ T cell subsets, play central roles in disease pathogenesis (**Figure 1**). Th1 cells, driven by IL-12, differentiate to secrete interferon-gamma (IFN-γ) and TNF-α, which orchestrate a Th1-type immune response by activating macrophages and enhancing antigen presentation (5). Conversely, Th17 cells arise from CD4+ T cells under the influence of TGF-β1 and IL-6, with their specific cytokine profile regulated by the transcription factor RORγt (62). These two cell types synergize in psoriasis, establishing a positive feedback loop that sustains inflammation. Cytokines secreted by Th1 and Th17 cells, including TNF-α, IFN-γ, IL-2, IL-6, IL-22, and IL-23, serve as potential disease biomarkers while directly promoting keratinocyte hyperproliferation, neutrophil infiltration, and the maintenance of a chronic inflammatory microenvironment (6, 8). The STAT3 gain-of-function (GOF) mouse model demonstrates that Th1/Th17 cell expansion, coupled with a reduction in skin regulatory T cell (Treg) populations, disrupts immune homeostasis, driving chronic inflammation (63).

##### 2.3.2.2 γδ T cells

The pathogenesis of psoriasis is intricately tied to aberrant γδ T cell activation. As non-classical T cells, γδ T cells arise from thymic development, migrating to peripheral tissues, or through local proliferation to maintain tissue homeostasis (7). In psoriasis, they contribute through two primary mechanisms: direct cytotoxicity against epidermal cells via perforin and granzyme release, and amplification of inflammation through secretion of pro-inflammatory cytokines, such as IFN-γ, IL-17, and IL-22, across multiple signaling pathways (5, 60). Under normal conditions, epidermal γδ T cells collaborate with Langerhans cells to uphold skin barrier homeostasis, but in psoriasis, this balance is disrupted, compromising immune surveillance (6, 25). In psoriasis-like mouse models, γδ T cells interact with cutaneous nerve fibers, with IL-17 production regulated by the sympathetic norepinephrine-β1-adrenergic receptor axis; inhibiting this pathway significantly attenuates inflammation (64). Metabolic dysregulation further exacerbates disease progression: high-cholesterol diets generate oxidized steroids that activate γδ T cells via GPR183 receptors, promoting IL-17 secretion and linking obesity to psoriasis severity (65).

##### 2.3.2.3 Regulatory cells

Dysfunction and phenotypic plasticity of regulatory T cells are pivotal in the pathogenesis of psoriasis. Tregs are broadly classified into thymic-derived natural Tregs (nTregs) and peripherally induced Tregs (iTregs), both reliant on the transcription factor FOXP3 to maintain their immunosuppressive functions (66, 67). In psoriatic lesions, Treg numbers are diminished, and their functionality is compromised, as evidenced by reduced FOXP3 expression and upregulated IL-17A production (68). Within the inflammatory microenvironment, some Tregs undergo a phenotypic shift, transforming into RORγt-expressing Foxp3+ IL-17+ cells that lose immunosuppressive capacity and instead promote keratinocyte hyperproliferation and neutrophil infiltration through IL-17A secretion, perpetuating a vicious inflammatory cycle (56, 68). In imiquimod (IMQ)-induced mouse models, Treg recruitment to inflammatory sites is impaired, and their clonal expansion is significantly diminished (68). Obesity, a key risk factor, exacerbates inflammation by affecting skin specific PPARγ+ Treg subpopulations (69). These Tregs, which possess anti-inflammatory properties, are reduced in abundance, triggering cutaneous lipotoxicity, oxidative stress, and mitochondrial dysfunction, thereby driving disease progression (69). Clinical data reveal a markedly lower peripheral blood Treg-to-Th17 ratio in psoriasis patients. Following umbilical cord mesenchymal stem cell (MSC) transplantation, Treg recovery correlates positively with reductions in Th17 and naive CD4+ T cell populations, as well as the degree of clinical remission, highlighting the therapeutic potential of modulating Treg function (70).

##### 2.3.2.4 CD8+ T cells

CD8+ T cells are central drivers of psoriasis pathogenesis, predominantly arising from skin-resident memory T cells (TRMs). Notably, αβ CD8+ T cell clones bearing psoriasis-specific antigen receptors that produce IL-17 persist in clinically resolved lesions, indicating their potential role as initiators of disease recurrence (58). Single-cell RNA sequencing has identified 11 transcriptionally diverse CD8+ T cell subsets in psoriatic lesions, with two Tc17 subsets significantly enriched. These subsets exhibit distinct yet developmentally related metabolic profiles and express the biomarker CXCL13, which correlates with disease severity (57, 71). CD8+ T cells surpass IL-17A+ CD4+ T cells as the primary source of IL-17A in lesions (3) and co-secrete IL-22, forming a distinctive Tc17/22-like cytokine profile (72). High expression of skin-homing receptors, such as CCR4 and cutaneous lymphocyte antigen (CLA), enables these cells to localize to the epidermis, where they continuously release pro-inflammatory mediators like IL-17A, driving aberrant keratinocyte proliferation and inflammatory mediator release (5, 72). A unique CD8+CCR10+ TRM subpopulation, observed in the circulation of patients with psoriatic arthritis, lacks cytotoxicity but exerts regulatory functions, with upregulated genes like RORC and IFNAR1 highlighting pathological distinctions from plaque psoriasis (72). Sustained CD8+ T cell activation relies on the CD69-LAT1-CD98 metabolic pathway and positive feedback from the IL-23/IL-17 axis (6, 73). The resulting cytokine network, encompassing IL-17A, IL-22, and IFN-γ, not only disrupts the epidermal immune microenvironment but also amplifies the inflammatory cascade by interacting with fibroblasts through MMP2-mediated CD100 shedding, forming a self-reinforcing inflammatory loop (74). This persistent immune response underscores CD8+ T cells as key effectors in the chronicity and relapse of psoriasis (8).

##### 2.3.2.5 Tissue-resident memory T cells

In psoriasis, TRM cells are critical pathogenic drivers due to their formation of an “inflammatory memory” following antigen exposure (58). Originating from infections or early inflammatory events, these cells are seeded in the skin, particularly in the sub-epidermis and hair follicle regions, following antigenic stimulation. This population includes CD69+ CD103+ TRM cells, which rely on IL-23 signaling for survival and function (57, 75). Th17-dominated TRM cells secrete high levels of pro-inflammatory cytokines, driving keratinocyte hyperproliferation and aberrant differentiation while inducing local chemokine production (e.g., CCL20). This attracts additional immune cells, such as dendritic cells and macrophages, to the lesion site, perpetuating a self-reinforcing inflammatory microenvironment (5, 72). Mechanistically, TRM cells rapidly respond to local pathogens or environmental triggers, such as fungal antigens, independently of circulating T cell recruitment, amplifying pro-inflammatory signals through cytokine release (5). Critically, these cells persist in the skin even after treatment-induced lesion resolution, remaining latent and capable of reactivating inflammation upon treatment cessation. This explains the characteristic *in situ* recurrence of psoriatic lesions (58, 72).

### 2.4 Cytokine networks and signaling pathways

#### 2.4.1 IL-17A/F

IL-17A and IL-17F, members of the interleukin-17 family, are pivotal in psoriasis pathogenesis (76). IL-17A, originally termed CTLA-8, is primarily secreted by Th17 cells, with additional contributions from CD8+ T cells, γδ T cells, natural killer (NK) cells, neutrophils, mast cells, and macrophages, underscoring their critical roles in immune regulation (5, 77). These cytokines are implicated in various inflammatory and autoimmune skin disorders, including psoriasis, atopic dermatitis, vitiligo, systemic lupus erythematosus, and malignant melanoma (76). Encoded at the same genetic locus (6p12), IL-17A and IL-17F share regulatory mechanisms and can form heterodimers. While IL-17A has been extensively studied for its role in autoimmunity, IL-17F, often expressed at higher levels in psoriatic lesions, has been relatively underappreciated (78). IL-17A is predominantly produced by IL-23R+ Th17 cells, whereas IL-17F is more commonly expressed by IL-23R- cells, such as mucosal-associated invariant T (MAIT) cells (71, 79). This differential expression highlights the superior efficacy of dual IL-17A/F inhibition over IL-17A-specific blockade (78). IL-17A and IL-17F activate NF-κB and MAPK signaling pathways through specific receptor complexes (IL-17RA/IL-17RC/IL-17RD), with key mediators including CARMA2, ACT1, and TRAF6. This signaling drives epidermal cells to overexpress inflammatory mediators, promoting immune cell recruitment and activation (80, 81). Notably, IL-17A induces keratinocytes to produce IL-23, a critical driver of Th17 cell differentiation and maintenance. Activated Th17 cells, in turn, secrete abundant IL-17A, IL-17F, and other cytokines, forming a self-amplifying feedback loop: keratinocyte-derived IL-23 sustains Th17 cell populations, while IL-17 stimulates further production of inflammatory mediators and IL-23, perpetuating chronic inflammation (6, 8). This cycle is a cornerstone of persistent inflammation in psoriasis. *In vitro* studies, often utilizing models like cultured keratinocytes, simplify IL-17 signaling analysis, primarily through the IL-17RA/IL-17RC receptor complex. However, IL-17RA-deficient mouse models reveal that psoriasis-like lesions persist, suggesting compensatory roles for other receptors, such as IL-17RC or IL-17RD (80, 82). While *in vitro* models enable precise variable control for drug screening (5, 14, 83), they fail to fully replicate the complex *in vivo* immune microenvironment, potentially underestimating inflammation or reducing physiological relevance. In contrast, *in vivo* models, such as imiquimod (IMQ)-induced psoriasis in mice, demonstrate that IL-17A signaling ablation significantly reduces skin inflammation, allowing direct assessment of disease phenotypes and therapeutic efficacy (e.g., FXYD3 deletion mitigates severity) (80, 84). However, interspecies differences limit result generalizability, necessitating validation with larger sample sizes. Advanced single-cell RNA sequencing of human psoriatic lesions has identified IL-17A+IFN-γ+ and IL-17F+IL-10- T cell subsets as potentially pathogenic populations (71). Although this approach resolves cellular heterogeneity, it struggles to dynamically monitor signaling pathway activation.

#### 2.4.2 IL-23

IL-23, a member of the IL-12 cytokine family, shares the p40 subunit with IL-12 but is distinguished by its unique p19 subunit, which specifically binds the IL-23 receptor to activate downstream signaling pathways (85). Primarily secreted by immune cells, such as dendritic cells (including plasmacytoid DCs) and T cell subsets, IL-23 is also produced by keratinocytes in psoriasis, though only immune cell-derived IL-23 holds pathological significance (8). This cytokine plays a central role in immune-mediated chronic inflammatory diseases, including psoriasis, psoriatic arthritis, Crohn’s disease, and uveitis, exerting broad immunopathologic effects (86). In psoriasis, IL-23 drives pathogenesis primarily through the IL-23/IL-17 signaling axis (5, 8), while also promoting neutrophil polarization via STAT3-RORγt and BATF pathways, among other mechanisms (55, 87). These actions enhance the secretion of pro-inflammatory mediators, intensifying local inflammation. In mouse models, intradermal IL-23 injection directly induces psoriasiform dermatitis, recapitulating the chronic inflammatory features of human psoriasis (88). However, IL-23 inhibition, while effective in reducing pathogenic Th17/Tc17 cells, fails to fully capture the clinical heterogeneity of patient populations, highlighting limitations of these models (78, 83). In contrast, single-cell RNA sequencing (scRNA-seq) studies of human psoriatic skin provide high-resolution insights, confirming that IL-23 inhibition rapidly downregulates JAK/STAT signaling and IL-23/Th17 pathway-related gene expression in keratinocytes (71, 89). However, small or heterogeneous sample sizes, such as studies analyzing only four samples post-IL-17A blockade, may introduce bias and underestimate the contributions of other IL-17 family members (90). Current research often emphasizes the IL-23/IL-17 axis, potentially overlooking other pathways, such as IL-1 or IL-36 signaling (91). IL-23 blockade reduces Th17/Tc17 cell frequency but does not fully normalize all inflammatory mediators, indicating that psoriasis involves complex, multifactorial networks, including IL-25 and prostaglandin E (PGE) signaling (55, 92). Longitudinal studies, such as early mechanistic analyses of IL-23 inhibitors, track biomarker dynamics effectively but are limited by short-term follow-ups (e.g., days post-treatment), which may miss long-term relapse effects and underexplore IL-23’s protective roles in the skin (89).

#### 2.4.3 TNF-α

Tumor necrosis factor-alpha (TNF-α), a pleiotropic pro-inflammatory cytokine of the TNF superfamily, is primarily secreted by immune cells, including dendritic cells, macrophages, T cells, and keratinocytes (5). It plays a critical role in the pathogenesis of various autoimmune diseases, such as psoriasis, rheumatoid arthritis, inflammatory bowel disease, Alzheimer’s disease, and multiple sclerosis (93). In psoriasis, TNF-α binds to tumor necrosis factor receptor 1 (TNFR1/TNFRSF1A) and receptor 2 (TNFR2), activating downstream NF-κB and MAPK signaling pathways (94). NF-κB signaling promotes the release of pro-inflammatory mediators, while the MAPK pathway drives keratinocyte hyperproliferation (95). Additionally, TNF-α enhances its own signaling by downregulating phosphoglycerate dehydrogenase (PHGDH), which triggers keratinocytes to release inflammatory mediators, such as IL-36γ, exacerbating skin barrier disruption (5). Notably, TNFR1 and TNFR2 exert opposing effects in psoriasis. In imiquimod-induced mouse models, TNFR1 knockout significantly reduces skin inflammation (96), whereas TNFR2 deletion increases neutrophil infiltration and IL-23 expression, worsening disease progression (97). These divergent outcomes may stem from differences in model induction (e.g., imiquimod stimulation versus knockout strategies) or the timing of inflammatory stage assessments. TNF-α inhibitors can paradoxically induce eczema, with affected patients showing upregulation of the TNF/IFN-γ signaling pathway (98). However, variations in risk among inhibitors (e.g., infliximab versus etanercept) remain unclear, potentially overlooking drug-specific effects. Systematic reviews of real-world cases provide insights into clinical characteristics (e.g., age of onset, time to onset) and treatment responses in TNF-α inhibitor-induced psoriasis (99, 100), but reliance on published cases risks reporting bias. Prospective molecular studies, such as those analyzing TNF-α response phosphorylation via flow cytometry, offer dynamic data but are limited by small sample sizes (e.g., n=25), which may compromise statistical robustness (101).

#### 2.4.4 IL-36

IL-36, a member of the IL-1 cytokine family, is predominantly secreted by keratinocytes in the skin, with additional contributions from dendritic cells, macrophages, endothelial cells, and dermal fibroblasts (102). This family comprises three pro-inflammatory agonists (IL-36α, IL-36β, IL-36γ) and one receptor antagonist (IL-36Ra), which collectively play pivotal roles in inflammatory diseases such as psoriasis (notably generalized pustular psoriasis [GPP] and palmoplantar pustulosis [PPP]), atopic dermatitis, hidradenitis suppurativa, Netherton’s syndrome, inflammatory bowel disease, and idiopathic pulmonary fibrosis (103). IL-36 drives inflammatory cascades by activating the IL-36R-mediated NF-κB signaling pathway (104). Keratinocyte-derived IL-36γ promotes chemokine release, such as IL-8, facilitating neutrophil recruitment while suppressing epidermal differentiation genes (e.g., keratinized bridging granule proteins), leading to epidermal barrier disruption and pustule formation (104, 105). Additionally, IL-36 forms a positive feedback loop with IL-23, amplifying inflammation (106). In GPP, overactivation of IL-36 signaling is frequently linked to IL36RN gene mutations, which impair IL-36Ra function, resulting in unopposed IL-36R downstream signaling (107, 108). Current research on IL-36 faces several limitations. Most studies rely on imiquimod-induced mouse models to mimic plaque psoriasis, which inadequately replicate the complexity of human GPP, while *in vitro* keratinocyte models fail to capture immune microenvironment interactions (91). The interplay between the IL-23/IL-17 axis and IL-36 signaling varies: some studies emphasize IL-23’s role in driving plaque-type lesions via Th17 cells, with IL-36 acting more directly on keratinocytes and neutrophils (109), whereas emerging evidence suggests keratinocyte-derived IL-23 directly regulates IL-36, indicating underappreciated cell-specific signaling differences (110). Therapeutically, anti-IL-36R antibodies demonstrate significant efficacy in GPP (111, 112), but data on their effectiveness in plaque psoriasis remain limited, fueling debate over whether IL-36 plays a minor role in plaque phenotypes or exhibits redundant effects with IL-17. Methodologically, most studies broadly reference IL-36 without distinguishing subtypes (113) and rely on knockout mouse models (113), often overlooking human immune heterogeneity.

#### 2.4.5 Other cytokines

Cytokines such as interferon-gamma (IFN-γ), granulocyte-macrophage colony-stimulating factor (GM-CSF), and phosphodiesterase-4 (PDE-4) play significant roles in the pathogenesis of psoriasis, contributing to the complex inflammatory network that sustains the disease. IFN-γ, markedly overexpressed in psoriatic lesions, is a key driver of Th1 immune responses, promoting chemokine production that amplifies local inflammation and facilitates immune cell infiltration (5). It enhances the expression of pro-inflammatory mediators, such as CXCL10, which recruit T cells and exacerbate cutaneous inflammation (8). However, clinical trials targeting IFN-γ alone have shown limited efficacy (114), underscoring the multifactorial nature of psoriasis, where single-molecule inhibition often fails to achieve comprehensive symptom control due to redundant inflammatory pathways.

GM-CSF, elevated in memory T cells within psoriatic lesions, potentiates the inflammatory effects of the IL-23/Th17 axis by activating neutrophils and macrophages (91, 115). Its role in amplifying inflammation involves enhancing myeloid cell function and promoting the release of pro-inflammatory cytokines, such as IL-6 and TNF-α, which further perpetuate the inflammatory cascade (116). However, GM-CSF expression depends on synergistic interactions with multiple cytokines, suggesting it functions as a cooperative rather than an independent driver of inflammation. This interdependence may limit the therapeutic efficacy of GM-CSF-targeted therapies, as blocking it alone may not disrupt the broader inflammatory network (117). PDE-4, a member of the phosphodiesterase family, is highly expressed in psoriatic skin and contributes to inflammation by degrading cyclic adenosine monophosphate (cAMP). This reduction in cAMP activates the cAMP-PKA-CREB-SIRT1 signaling pathway, promoting the production of inflammatory mediators, including IL-17 and IL-23 (117). In preclinical psoriasis models, PDE-4 inhibition significantly reduces epidermal thickening and inflammatory markers, demonstrating potent local anti-inflammatory effects (118). Clinically, PDE-4 inhibitors, such as apremilast, have shown efficacy in reducing plaque severity and improving patient outcomes, though their benefits are often partial, indicating the need for combination therapies to address the multifaceted inflammatory milieu of psoriasis (119). These findings highlight the complementary roles of IFN-γ, GM-CSF, and PDE-4 in sustaining psoriatic inflammation and the challenges of targeting individual molecules within a complex cytokine network.

### 2.5 Insights from omics and experimental models

Omics technologies have revolutionized psoriasis research by unveiling complex immune interactions and identifying precise therapeutic targets. Genomics and transcriptomics have pinpointed critical hub genes, such as IL17A and IL1B, central to the IL-23/IL-17 axis that drives T cell-mediated inflammation (3, 71). Proteomics reveals altered protein profiles, while single-cell RNA sequencing (scRNA-seq) elucidates cellular heterogeneity, uncovering dysregulated pathways in keratinocytes and T cells (27, 120). These approaches have also identified biomarkers for disease stratification and therapeutic monitoring, advancing the frontier of personalized medicine. Experimental models complement these molecular insights. Imiquimod-induced mouse models recapitulate psoriasis-like inflammation through TLR7 activation, triggering the IL-23/IL-17 axis (78). *In vitro* keratinocyte cultures and skin organoids validate key therapeutic targets, such as the IL-23/IL-17 pathway, facilitating robust preclinical assessments (83, 121). Despite limitations in capturing the chronicity of human psoriasis, these models effectively bridge molecular discoveries to therapeutic innovation, driving the development of treatments like secukinumab. The future integration of multi-omics strategies with advanced models, such as humanized mice, holds immense potential to refine precision therapies for psoriasis, enhancing clinical outcomes.

## 3 Psoriasis and microbiota

Emerging research highlights a significant association between psoriasis and the gut microbiota, revealing distinct compositional and functional differences in the intestinal flora of patients compared to healthy individuals. Psoriatic patients exhibit reduced microbial species richness, with notable enrichment of *Streptococcus* spp. and alterations in *Prevotella* spp., alongside decreased community diversity (122, 123). These differences persist independent of shared environmental factors, as confirmed by studies controlling for cohabiting partners (122). Animal models further demonstrate that gut dysbiosis directly exacerbates psoriasis-like skin inflammation. For instance, transplanting microbiota from mice with severe inflammation to those with milder symptoms significantly worsens skin lesions, underscoring a causal link (123).

**Figure 2:**
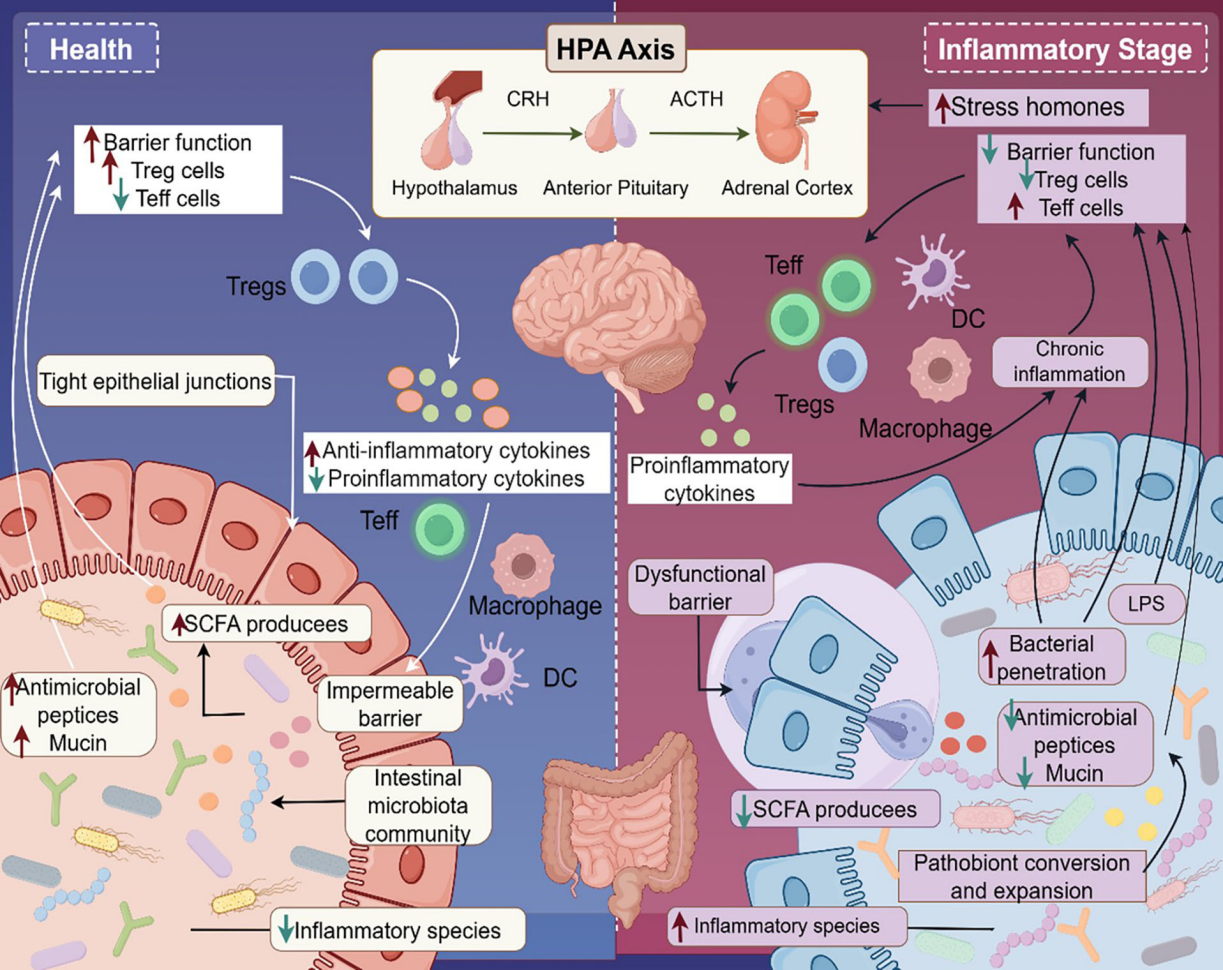
Gut-brain-skin axis in psoriasis pathogenesis. This figure illustrates the “gut-brain-skin axis” in psoriasis. In healthy conditions, the gut microbiota supports intestinal barrier integrity by producing short-chain fatty acids (SCFAs), which maintain tight junctions and antimicrobial peptide/mucin barriers. SCFAs also suppress pathogenic commensal bacteria and promote regulatory T cell (Treg) differentiation, fostering anti-inflammatory homeostasis. In contrast, chronic stress activates the hypothalamic-pituitary-adrenal (HPA) axis, triggering the release of corticotropin-releasing hormone (CRH), adrenocorticotropic hormone (ACTH), and stress hormones. This disrupts intestinal barrier function, promoting lipopolysaccharide (LPS) translocation and the release of pro-inflammatory cytokines (e.g., IL-6, TNF-α). These mediators drive effector T cell (Teff) and macrophage polarization, initiating systemic inflammation that exacerbates psoriatic skin lesions. This mechanism underscores the intricate interplay among intestinal barrier dysfunction, neuroendocrine stress, and cutaneous inflammation in psoriasis.

This microbial influence is partly mediated through host-microbiota metabolic interactions, with reduced short-chain fatty acid (SCFA) production, such as butyrate and propionate, playing a central role (124, 125). SCFAs, generated through microbial fermentation of dietary fiber, modulate psoriasis severity via three key pathways (**Figure 2**): 1) suppression of regulatory T cell (Treg) differentiation, leading to overactivation of the IL-23/Th17 axis (126); 2) disruption of intestinal barrier integrity, enabling translocation of lipids, polysaccharides, and other metabolites that trigger systemic inflammation (127); and 3) modulation of the gut-brain axis, influencing neurotransmitter production and indirectly regulating cutaneous immune responses (128). Given the pivotal role of the gut microbiome in psoriasis, dietary interventions offer a promising strategy to restore microbial balance and mitigate inflammation. The Mediterranean diet, rich in fiber, fruits, vegetables, and healthy fats, has been shown to enhance microbiome diversity and improve clinical outcomes in psoriasis (129). Beyond diet, targeted interventions such as prebiotics, probiotics, and microbiota-modulating therapies hold significant potential for both prevention and treatment by boosting SCFA production and fostering a balanced microbiome (130, 131).

However, current evidence is largely derived from animal models or small-scale observational studies, with a paucity of high-quality clinical trials validating these interventions in psoriasis populations. Individual variability in microbiome responses to dietary changes, coupled with differences across psoriasis subtypes (e.g., plaque versus psoriatic arthritis), remains underexplored, necessitating stratified analyses in future studies. The complex interplay among diet, microbiota, and immunity warrants investigation within a systems biology framework to fully elucidate these relationships.

Emerging therapeutic strategies aim to disrupt this pathogenic cycle through innovative approaches. These include fecal microbiota transplantation to restore microbial equilibrium (132, 133), engineered microbes designed to enhance Treg cell populations (134), and precision delivery of anti-inflammatory metabolites via nanoparticle-loaded bacterial vesicles (135). Nonetheless, the predominantly cross-sectional nature of existing studies limits causal inferences. Robust longitudinal cohort studies are urgently needed to establish temporal associations and clarify the roles of specific microbial strains and their metabolites in psoriasis pathogenesis and progression.

## 4 Psoriasis and neuroimmunology

**Figure 3:**
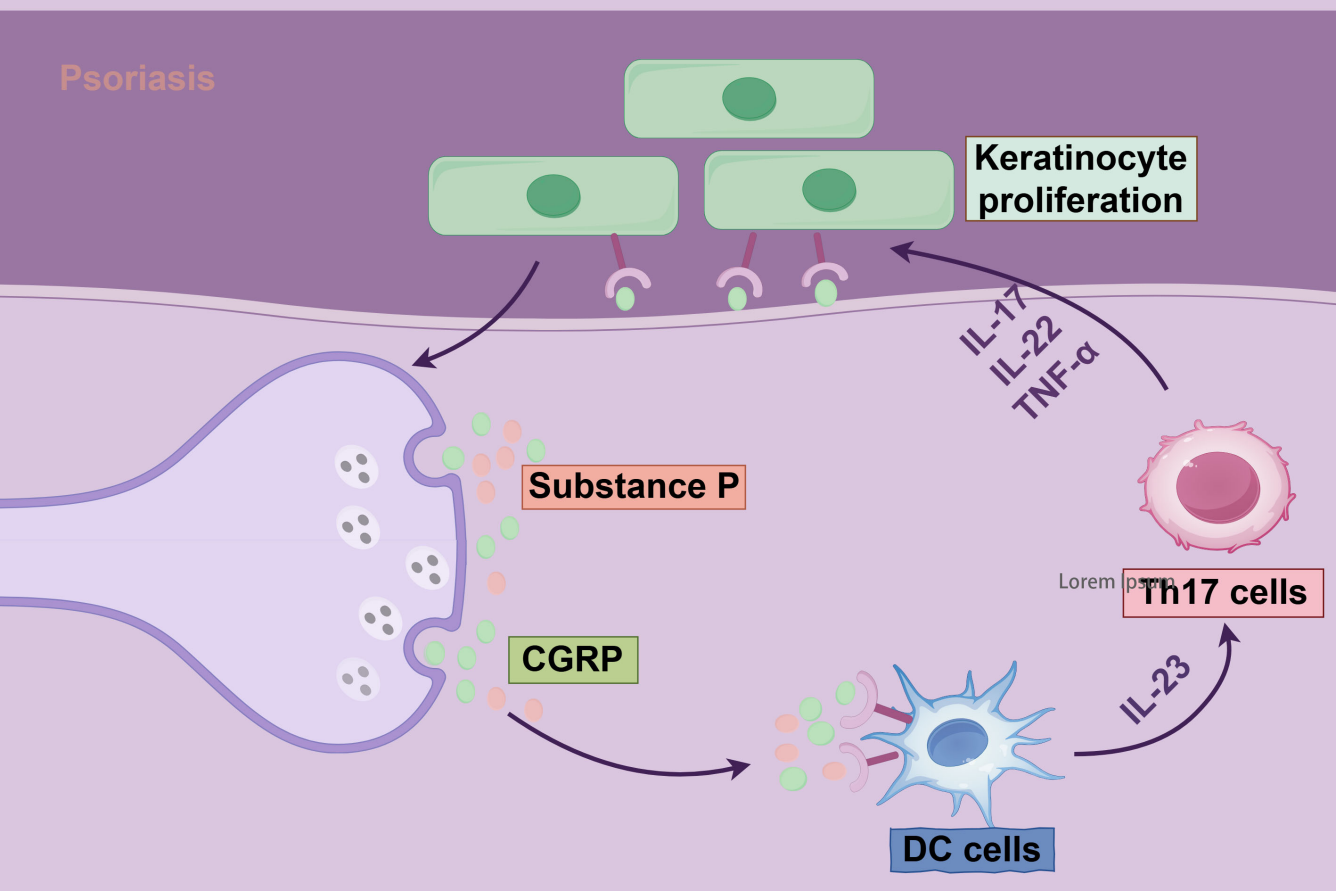
Mechanisms of Th17 cells and neuropeptide CGRP in keratinocyte proliferation in psoriasis. The diagram illustrates the pivotal role of the nervous system in psoriasis pathogenesis. Through the production of neuropeptides and neurotransmitters, coupled with systemic or localized changes in chemical mediators and their receptors, the nervous system engages in dynamic interactions with immune cells and cytokines. These interactions drive pathological keratinocyte hyperproliferation and sustain an inflammatory microenvironment, hallmark features of psoriasis.

Neuroimmune interactions are pivotal in the pathogenesis of psoriasis, a chronic inflammatory skin disorder (136, 137). The nervous system engages in bidirectional communication with immune cells through neurotransmitters and neuropeptides, such as calcitonin gene-related peptide (CGRP) and substance P, directly modulating cutaneous inflammation (**Figure 3**) (138, 139). Sensory neuron-derived neurotransmitters stimulate immune cell receptors, driving keratinocyte hyperproliferation and Th17 cell differentiation while amplifying IL-17 production, thus establishing a self-reinforcing neuroimmune feedback loop (5, 11). This interplay is central to hallmark psoriatic features, including epidermal thickening, neutrophil infiltration, and pruritus. Neuroimmune dysregulation also underlies neurological comorbidities associated with psoriasis (140, 141). Robust clinical evidence, with nine of eleven studies confirming the link, indicates a significantly increased risk of mild cognitive impairment and dementia (91). Proposed mechanisms suggest that systemic inflammation breaches the blood-brain barrier, with cytokines such as IL-17 and TNF-α directly affecting neurons and glial cells, triggering neuroinflammation and synaptic dysfunction (142). Furthermore, the skin-brain axis posits that chronic pruritus and aberrant neural signaling, induced by mechanical stress, exacerbate systemic inflammation through activation of the hypothalamic-pituitary-adrenal (HPA) axis (139, 142). However, the precise contribution of this pathway in humans awaits further validation.

At the molecular level, sensory neuron-specific acid-sensing ion channel 3 (ASIC3) plays a critical role in mediating neurogenic inflammation (136). Deletion of ASIC3 in preclinical models reduces psoriasiform lesions, whereas CGRP supplementation reinstates inflammation, highlighting the essential role of neurotransmitter-immune cell crosstalk (136, 143). Fibroblast subsets further enhance these interactions by facilitating synapse formation with IL-17-producing γδ T cells (91). These findings inform innovative neuroaxis-targeted therapeutic strategies, including neurotoxin-mediated signaling blockade and neurotransmitter receptor modulation (136, 139, 144).

Psoriasis shares neuroimmune pathways with comorbidities such as inflammatory bowel disease and depression (145, 146). Although biologics effectively alleviate cutaneous inflammation, their limited impact on neurological symptoms and pruritus underscores the need for integrated therapies targeting both immune and nervous systems (12, 15). Future investigations employing single-cell multi-omics approaches will further dissect the cutaneous neuroimmune microenvironment, identifying precise therapeutic targets to address the complex, multifaceted pathology of psoriasis.

## 5 Translational research: from bench to bedside in psoriasis

Translational research in psoriasis converts mechanistic discoveries into clinical advancements, emphasizing biomarker-driven precision medicine. Cytokine profiles (e.g., IL-17, TNF-α), T-cell subsets like Th17 frequencies, and genetic variants such as HLA-C*06:02 enable precise evaluation of disease severity, prediction of biologic response, and long-term monitoring (6, 147). Obtained from blood or skin biopsies, these biomarkers facilitate personalized treatment and early intervention. Psoriasis-related systemic inflammation, originating in the skin microenvironment, contributes to comorbidities like cardiovascular disease, metabolic syndrome, psoriatic arthritis, and depression through shared TNF-α/IL-17 pathways that drive atherosclerosis, insulin resistance, and neuroinflammation (148–151). Preclinical efforts are advancing nano-therapeutics, with nanoparticle-based delivery systems targeting multiple cytokines and immune cells within the epidermal immune microenvironment. These systems enhance drug penetration, reduce off-target effects, and remodel inflammatory milieus, improving outcomes in refractory cases (152, 153).

**Figure 4:**
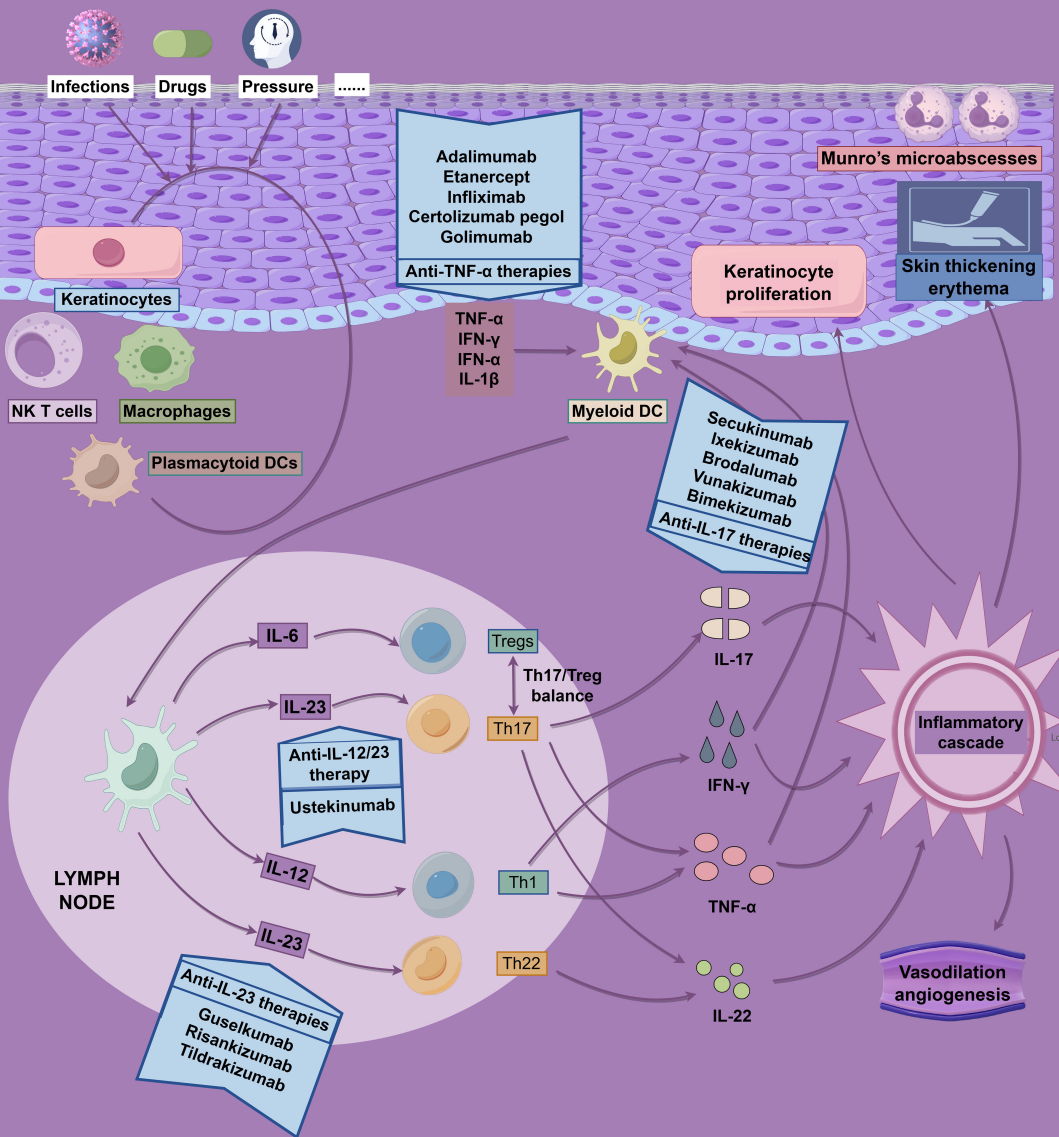
Pathophysiology and drug targets of psoriasis. The pathophysiology of psoriasis is driven by dysregulated, self-perpetuating activation of the adaptive immune system, characterized by a complex interplay of inflammatory pathways. This figure employs a dual-axis format (“inflammatory cascade - targeted intervention”) to integrate and illustrate the core pathological mechanisms of psoriasis alongside corresponding therapeutic strategies.

The IL-23/Th17 axis is central to psoriasis pathogenesis. Monoclonal antibodies targeting IL-23p19 (e.g., guselkumab, risankizumab, tildrakizumab) and IL-17 (e.g., secukinumab, ixekizumab, brodalumab) demonstrate robust efficacy in clinical trials (**Supplementary Table 1**; **Figure 4**) (154). The GUIDE trial (NCT03895112) showed that guselkumab’s p19 subunit blockade reshapes the skin’s immune milieu, maintaining efficacy with extended dosing intervals (Q8W to Q16W) and promoting microenvironmental homeostasis through early intervention (155). Risankizumab exhibited consistent short- and long-term efficacy with a favorable safety profile in Phase III trials (156). Single-cell transcriptomics revealed CD8^+^ T-cell regression within 3 days of IL-23 blockade, alongside myeloid remodeling and IFN-γ downregulation by day 14 (157). However, IL-23 inhibitors do not fully restore epidermal immune homeostasis despite reducing Th17/Tc17 cell frequencies, suggesting a role for other factors, such as IL-12, in maintaining barrier function (158).

Biosimilars, such as Amjevita and Imraldi, match adalimumab’s efficacy in real-world studies, supporting their cost-effective integration (159). Bimekizumab, a dual IL-17A/F inhibitor, effectively suppresses neutrophil-associated gene modules but is less effective at regulating epidermal metabolism genes compared to IL-23 inhibitors (160, 161). Its Phase III trial confirmed concurrent improvements in depressive symptoms (PHQ-9 scale), highlighting skin-neuroimmune interactions (162). Newly approved monoclonal antibodies, vunakizumab (IL-17) and xeligekimab (IL-23), further expand therapeutic options (163, 164). Despite these advances, 25–50% of patients exhibit biologic resistance, underscoring the complexity of the immune microenvironment and the need for individualized therapies (165).

Oral therapies are reshaping psoriasis management. Apremilast, a PDE4 inhibitor, demonstrates efficacy across diverse psoriasis types, including specialty sites (e.g., scalp, nails) and pediatric patients, with mild, transient side effects (e.g., nausea, diarrhea, headache) and low discontinuation rates (119, 166, 167). Its oral administration eliminates the need for routine laboratory monitoring, enhancing patient convenience and adherence, particularly for those with comorbidities or in resource-limited settings (168). However, optimizing long-term efficacy and minimizing discontinuation remain priorities. TYK2 inhibitors, such as deucravacitinib (BMS-986165) and zasocitinib, show significant efficacy in plaque psoriasis in Phase II trials, with deucravacitinib achieving PASI 75 in 53–67% of patients and a favorable safety profile (169, 170). Zasocitinib’s Phase IIb trial (NCT04999839) reported dose-dependent PASI 75 responses (44–68% at 5–30 mg), with Phase III ongoing (171). Long-term safety data for TYK2 inhibitors are still needed to confirm their role in routine care.

Tapinarof, a first-in-class, nonsteroidal, topical aryl hydrocarbon receptor (AhR) agonist, represents a breakthrough in topical psoriasis management. It exerts therapeutic effects by downregulating pro-inflammatory cytokines (e.g., IL-17A, IL-17F), promoting skin barrier restoration through upregulation of key proteins like filaggrin and loricrin, and mitigating oxidative stress via the nuclear factor erythroid-2-related factor 2 (NRF2) pathway (172, 173). Its multifaceted mechanism addresses both inflammation and barrier dysfunction, offering a promising alternative for patients seeking non-systemic options.

Preclinical studies targeting epidermal Peli1 reduced IL-17A production by skin-resident T17 cells, improving psoriasis-like dermatitis (174). Delivery of STAT3 siRNA via dendritic lipopeptide nanocarriers penetrated the skin barrier, reducing epidermal thickness and restoring immune homeostasis in mouse models, though translation to human applications requires further validation due to differences between murine and human disease (175). Machine learning-based mRNA prediction models, trained on 1,145 samples, achieve >85% accuracy in forecasting drug responses, but sample heterogeneity and computational limitations hinder comprehensive biological representation (176). Emerging targets, such as IL-21 for Th17/Treg modulation and microbial influences on IL-23/IL-17 signaling, offer promising avenues for overcoming resistance (177).

## 6 Challenges and future directions in psoriasis immunotherapy

### 6.1 Limitations of current immunotherapy

Despite significant progress in psoriasis immunotherapy, substantial challenges persist across multiple domains. Approximately 25–50% of patients exhibit suboptimal responses or complete resistance to IL-17/IL-23-targeted biologics (178), reflecting an incomplete understanding of the disease’s complex immunopathological landscape. High relapse rates following treatment discontinuation necessitate lifelong adherence, raising long-term safety concerns (179). Immunosuppression-related complications, such as fungal infections affecting ~12.3% of biologic users, indicate disruptions in cutaneous microbial homeostasis (179). Additionally, immune checkpoint inhibitor (ICI) therapy in oncology patients increases psoriasis risk (hazard ratio 2.43) through aberrant T-cell modulation (180).

Metabolic comorbidities significantly impair therapeutic efficacy. In obese or dyslipidemic patients, free fatty acid-induced neutrophil extracellular traps (NETs) amplify γδ T17-driven IL-17 inflammation, reducing the effectiveness of IL-17A inhibitors (181). The limited capacity of IL-23 inhibitors to modulate IL-17F-expressing T17 subsets contributes to resistance (182). Genetic studies reveal that known genome-wide association study (GWAS) risk loci account for only a portion of heritability, suggesting undiscovered regulatory genes influence treatment outcomes (183). Current therapies often focus on adaptive immunity, overlooking innate immune dysregulation, such as Gasdermin E-mediated keratinocyte pyroptosis and FGF12-driven cell cycle abnormalities, which exert lymphocyte-independent pathogenic effects unaddressed by biologics (184, 185). Economic barriers further complicate access, with high-efficacy agents like xeligekimab [achieving 74.4% PASI90 responses (164)] often exceeding insurance coverage limits. The absence of predictive biomarkers for IL-17/IL-23 inhibitors leads to trial-and-error prescribing, underscoring the need for more tailored approaches.

### 6.2 Therapeutic advancements and future directions

Complementing the IL-23/IL-17 axis, the STING pathway amplifies inflammation by activating dendritic cells to produce IL-17 and IFN-γ (186). Recent discoveries highlight neuroimmune interactions, with ASIC3 channels exacerbating inflammation via neurogenic pathways (143) and sympathetic CaMKII-γ+ nerves driving pathogenesis through norepinephrine release (139). Therapeutic innovations, such as risankizumab’s selective modulation of pathogenic type 17 T-cell subsets (187) and STING inhibition to reduce IL-17A production (186), are expanding treatment options. Novel strategies leverage advanced delivery systems and cellular therapies. Dendritic lipopeptide-based transdermal siRNA delivery offers non-invasive interventions (188), while engineered mesenchymal stem cell (MSC)-derived extracellular vesicles address metabolic and immunological imbalances (189). Molecular studies identify BTK and MMP-9 as regulators of NLRP3 inflammasome responses (190) and MMP2-high fibroblasts as modulators of CD8+ T-cell residency via CD100 interactions (191), broadening therapeutic horizons beyond traditional biologics.

Insights into comorbidities reveal that saturated fatty acids exacerbate NETosis in obesity-associated psoriasis (181), while disrupting macrophage-platelet feedback loops enhances efferocytosis (49). These findings highlight the limitations of current biologics in addressing systemic effects, advocating for combinatorial therapies. Notably, keratinocyte ferroptosis drives systemic inflammation, with inhibitors like liproxstatin-1 showing efficacy comparable to IL-12/IL-23/TNF-α biologics (192). Fibroblasts sustain inflammatory niches through metalloproteinase-mediated interactions with TRM cells (193).

TRM-targeted therapies, such as STAT3 inhibitors delivered via skin-penetrating dendritic lipopeptide nanoparticles, demonstrate promising efficacy (194). Emerging modalities include MSC-derived extracellular vesicles (195) and nanomaterial-based co-delivery systems integrating immune checkpoint inhibitors with cytokine modulators (196, 197). Biosimilars like SB17 improve accessibility by matching ustekinumab’s efficacy (198), though cardiovascular risks associated with biologics support on-demand regimens (199, 200). These advancements address neuroimmune dynamics (144, 201), metabolic perturbations (202, 203), and innovative delivery mechanisms (195, 204), advancing precision medicine.

## 7 Conclusion

The future of psoriasis management will center on integrating precision-driven, innovative, and individualized therapeutic strategies. Despite significant challenges—such as biologic resistance, high relapse rates, economic barriers, and the intricate interplay of innate and adaptive immune dysregulation alongside comorbidities—emerging solutions are promising. Novel therapies targeting pathways like STING and neuroimmune interactions, coupled with advanced delivery systems, including nanoparticle-based siRNA and MSC-derived extracellular vesicles, pave the way for more effective interventions. Combinatorial approaches, supported by predictive biomarkers and personalized treatment frameworks, will enhance therapeutic precision, optimize patient outcomes, and alleviate healthcare burdens in psoriasis management.

## Glossary

ACT1: NF-κB Activator 1
ACTH: Adrenocorticotropic Hormone
AhR: Aryl Hydrocarbon Receptor
ASIC3: Acid-Sensing Ion Channel 3
BATF: Basic Leucine Zipper ATF-Like Transcription Factor
BTK: Bruton’s Tyrosine Kinase
cAMP: Cyclic Adenosine Monophosphate
CARMA2: Caspase Recruitment Domain Family Member 2
CCL: Chemokine (C-C motif) Ligand
CCR: Chemokine Receptor
CD: Cluster of Differentiation
CGRP: Calcitonin Gene-Related Peptide
CLA: Cutaneous Lymphocyte Antigen
CREB: cAMP Response Element-Binding Protein
CRH: Corticotropin-Releasing Hormone
CXCL: Chemokine (C-X-C motif) Ligand
CXCR4: C-X-C Chemokine Receptor Type 4
DCs: Dendritic Cells
EIME: Epithelial Immune Microenvironment
FOXP3: Forkhead Box P3
FXYD3: FXYD Domain Containing Ion Transport Regulator 3
GM-CSF: Granulocyte-Macrophage Colony-Stimulating Factor
GPP: Generalized Pustular Psoriasis
GPR183: G Protein-Coupled Receptor 183
GWAS: Genome-Wide Association Studies
HLA-C06:02: Human Leukocyte Antigen C06:02
HPA: Hypothalamic-Pituitary-Adrenal
ICI: Immune Checkpoint Inhibitor
IL: Interleukin
IL36RN: Interleukin 36 Receptor Antagonist
ILCs: Innate Lymphoid Cells
IMQ: Imiquimod
JAK: Janus Kinase
KLRG1: Killer Cell Lectin-Like Receptor G1
LCs: Langerhans Cells
lncRNAs: Long Non-Coding RNAs
LPS: Lipopolysaccharide
MAIT: Mucosal-Associated Invariant T
MCPIP3: Monocyte Chemoattractant Protein-Induced Protein 3
MDSCs: Myeloid-Derived Suppressor Cells
MEG3: Maternally Expressed Gene 3
MMP: Matrix Metalloproteinase
MSC: Mesenchymal Stem Cell
NETs: Neutrophil Extracellular Traps
NF-κB: Nuclear Factor kappa-light-chain-enhancer of Activated B Cells
NFKBIA: Nuclear Factor of Kappa Light Polypeptide Gene Enhancer in B-Cells Inhibitor, Alpha
NLRP3: NOD-Like Receptor Pyrin Domain-Containing 3
NRF2: Nuclear Factor Erythroid-2-Related Factor 2
PASI: Psoriasis Area and Severity Index
PDE-4: Phosphodiesterase-4
Peli1: Pellino E3 Ubiquitin Protein Ligase 1
PGE: Prostaglandin E
PHGDH: Phosphoglycerate Dehydrogenase
PHQ-9: Patient Health Questionnaire-9
PKA: Protein Kinase A
PPARγ: Peroxisome Proliferator-Activated Receptor Gamma
PPP: Palmoplantar Pustulosis
PSORS: Psoriasis Susceptibility Loci
RORγt: Retinoic Acid-Related Orphan Receptor Gamma t
ROS: Reactive Oxygen Species
scRNA-seq: Single-Cell RNA Sequencing
SIRT1: Sirtuin 1
slanDCs: 6-Sulfo LacNAc Dendritic Cells
STAT3: Signal Transducer and Activator of Transcription 3
STING: Stimulator of Interferon Genes
TGR5: Takeda G Protein-Coupled Receptor 5
Th: T-helper
TLRs: Toll-Like Receptors
TNF-α: Tumor Necrosis Factor-alpha
TNFAIP3: Tumor Necrosis Factor Alpha-Induced Protein 3
TNFR1: Tumor Necrosis Factor Receptor 1
TNFR2: Tumor Necrosis Factor Receptor 2
TRAF6: TNF Receptor-Associated Factor 6
Treg: Regulatory T Cell
TRM: Tissue-Resident Memory T Cell
TYK2: Tyrosine Kinase 2
UC-MSC: Umbilical Cord Mesenchymal Stem Cells
UVB: Ultraviolet B
