# Supplementary material for: Skin immune microenvironment in psoriasis: from bench to bedside
Source: Front Immunol. 2025 Aug 29;16:1643418. doi: 10.3389/fimmu.2025.1643418 (PMC12426051; doi:10.3389/fimmu.2025.1643418)
Supplement: Supplementary file 1 [file Table1.docx]

**Supplement Table 1**  **Cytokine-Targeted Biologics for Psoriasis Therapy**

| **Drug Class** | **Systemic Agent** | **Molecular Structure** | **Regulatory Approvals** | **Efficacy Outcomes** | **Adverse Events** |
| --- | --- | --- | --- | --- | --- |
| Anti-TNF-α | Adalimumab | Human monoclonal IgG1 antibody | China: Moderate to severe PsO in adults Severe PsO in children aged 4 and above.  FDA: Adult PsO, PsA.  EMA: Adult PsO, PsA; PsO in children over 4 years old.  Japan: Adult PsO, PsA, PP | Adults: 71% achieve PASI 75 at week 16 (placebo, 7%)[1] | The duration of the treatment period was not sufficient to assess long-term efficacy and safety, and the trial did not include an active control consisting of other systemic therapies. |
|  | Etanercept | Fusion protein between a TNF-α receptor protein and the crystallizable fragment portion of IgG1 | China: Moderate to severe PsO in adults  FDA: PsO for adults and children over 4 years old; PsA in adults and children over 2 years old.  EMA: PsO for adults and children over 6 years old; PsA in adults and children over 12 years old; | Adults: 49% achieve PASI 75 at week 12 (placebo, 3%)[2];  Children (aged 4-17 years): 57% achieve PASI 75 at week 12(placebo, 11%)[3] | Nasopharyngitis, headache, elevated blood insulin, diarrhea, injection-site erythema, pharyngitis, arthralgia, injection-site reaction, fatigue, rash, nausea, anemia, mucositis, neutropenia, respiratory distress, lung infections and sepsis. |
|  | Infliximab | Human chimeric monoclonal IgG1 antibody | China: Severe PsO in adults  FDA/EMA: Adult PsO、 PsA；  Japan: Adult PsO, PsA, PP, PE | Adults: 80% achieve PASI 75 at week 10 (placebo, 3%)[4]; 55% achieve PASI 75 at week 50 (placebo/infliximab, 77%)[4] | Cellulitis of the cheeks, hepatitis, fatigue, nausea and loss of appetite, jaundice. |
|  | Certolizumab pegol | Pegylated humanized antibody fragment | FDA:Moderate to severe PsO and PsA in adults | Adults: 83% achieve PASI 75 at week 16 (placebo, 12%)[5] | Nasopharyngitis, upper respiratory tract infection, hypertension, mesenchymal oligodendroglioma,  basal cell carcinoma, breast cancer, clear cell renal carcinoma, glioblastoma, Hodgkin's disease, keratoacanthoma, diarrhea, headache and laryngeal carcinoma. |
|  | Golimumab | Fully humanized anti-TNFα monoclonal antibody | FDA：PsA for adults and children over two years old | Adult：75.1% achieve ACR20 at week 14(placebo, 21.8%)[6] | The most common ARDs is upper respiratory tract infections (such as nasopharyngitis, pharyngitis and laryngitis, etc.). |
| Anti-IL-17 | Secukinumab | Human monoclonal IL-17A antibody | China: Moderate to severe PsO in adults and children over 6 years old; Adult PsA.  FDA/EMA: PsO for adults and children over 6 years old; PsA in adults and children over 2 years old.  Japan: Adult PsO, PsA, PP; | Adults: 82% achieve PASI 75 at week 12 (placebo: 4%); 65%achieve IGA 0/1 at week 12(placebo, 2%)[7] | Nasopharyngitis, upper respiratory tract infection, oral or vulvovaginal candida infections, inflammatory bowel disease (ulcerative colitis). |
|  | Ixekizumab | Humanized monoclonal IL-17A antibody | China: Moderate to severe PsO in adults；  FDA: Adult PsO, PsA；PsO in children over 6 years old  EMA: Adult PsO, PsA:；PsO in children over 6 years old and weighing more than 25kg；  Japan: Adult PsO, PsA, PP, PE； | Adults: 90% achieve PASI 75 at week 12 (placebo, 2%); 83%achieve IGA 0/1 at week 12(placebo, 2%)[8] | Not Available |
|  | Brodalumab | Human monoclonal IL-17A receptor antibody | China/FDA: Moderate to severe PsO in adults. | Adults: 86% achieve PASI 75 at week 12 (placebo, 8%); 80% achieve IGA 0/1 at week 12(placebo, 4%)[9] | Nasopharyngitis, upper respiratory tract infection, headache, depression, Candida infection and arthralgia. |
|  | Vunakizumab | Humanized IgG1 subtype anti-IL-17A monoclonal antibody | China: Moderate to severe PsO in adults. | Adults: 76.8% achieve PASI 90 at week 12 (placebo, 0.9%); 71.8% achieve sPGA 0/1 at week 12(placebo, 0.4%)[10] | Upper respiratory tract infections and hyperuricemia. |
| Anti-IL-17A/F | Bimekizumab | Monoclonal IgG1 antibody | FDA/EMA:Moderate to severe PsO in adults;  Japan:Adult PsO, GPP, PE； | Adults: 86.2% achieve PASI 90 at week 16 (placebo, 4.8%); 85.3% achieve IGA 0/1 at week 16(placebo, 57.2%)[11] | Upper respiratory tract infection, nasopharyngitis, viral meningitis, colorectal polyps, colon cancer and fungal infections (oral candidiasis, oral fungal infections, vulvovaginal fungal infections, and tinea pedis). |
| Anti-IL-12/23 | Ustekinumab | Human monoclonal antibody against the p40 subunit, shared by IL-12/23 | China: Moderate to severe PsO in adults and children over 6 years old  FDA/EMA: Adult PsO, PsA; PsO in children over 6 years old  Japan: Adult PsO, PsA | Adults: 67% (45mg) and 76%(90mg) achieve PASI 75 at week 12(placebo, 4%)[12]；  Children (aged 12-17 years): 78% (45 mg) and 81%  78 (90 mg) achieve PASI 75 at week 12 (placebo, 11%)[13] | Headaches, nasopharyngitis, joint pain, high blood pressure and itching |
| Anti-IL-23 | Guselkumab | Human monoclonal IL-23 antibody | China: Moderate to severe PsO in adults；  FDA: Adult PsO, PsA；  EMA: Adult PsO, PsA；  Japan: Adult PsO, PsA, PP, PPP, PE； | Adults: 73% achieve PASI 90 at week 16 (placebo, 3%); 85% achieve IGA 0/1 at week 16 (placebo: 8%)[14] | Nasopharyngitis, upper respiratory tract infection, rectal adenocarcinoma, prostate cancer, plasma cell myeloma, melanoma in situ, nonfatal myocardial infarctions, nonfatal stroke and uveitis. |
|  | Risankizumab | Human monoclonal IL-23 antibody | FDA/EMA:Adult PsO、PsA；  Japan: Adult PsO, PsA, PE,PP； | Adults: 75% achieve PASI 90 at week 16 (placebo, 4%); 86% achieve IGA 0/1 at week 16(placebo, 7%)[15] | Nasopharyngitis, upper respiratory tract infection, oral or vulvovaginal  candida infections. |
|  | Tildrakizumab | Human monoclonal IL-23 antibody | China: Moderate to severe PsO in adults；  FDA/EMA/ Japan: Adult PsO | Adults: 64% achieve PASI 75 at week 12 (placebo, 6%); 58% achieve IGA 0/1 at week 12(placebo, 7%)[16] | Acute myocardial infarction, COVID-19 pneumonia, deep vein thrombosis,  hypertension, nasopharyngitis,  pharyngitis, rhinitis, urinary tract  infection, upper respiratory  tract infection. |
| Anti-IL-36R | Spesolimab | Humanized IgG1 monoclonal antibody | China/FDA: GPP attacks occur in adults and adolescents aged 12 and above with a weight >40kg.  EMA/ Japan: GPP attacks in adults | Adult：54% of the patients had no visible pustules after one week（placebo, 6%）；43% of the patients achieved skin clearance/near-clearance after one week（placebo, 11%）[17] | Urinary tract infections, influenza, folliculitis, otitis externa, upper respiratory tract infections, pustules, weakness, fatigue, nausea, vomiting, headache, itching, and infusion site hematoma and bruising. |

**References**

1. Menter, A., et al., Adalimumab therapy for moderate to severe psoriasis: A randomized, controlled phase III trial. J Am Acad Dermatol, 2008. 58(1): p. 106-15.
2. Papp, K.A., et al., A global phase III randomized controlled trial of etanercept in psoriasis: safety, efficacy, and effect of dose reduction. Br J Dermatol, 2005. 152(6): p. 1304-12.
3. Paller, A.S., et al., Etanercept treatment for children and adolescents with plaque psoriasis. N Engl J Med, 2008. 358(3): p. 241-51.
4. Reich, K., et al., Infliximab induction and maintenance therapy for moderate-to-severe psoriasis: a phase III, multicentre, double-blind trial. Lancet, 2005. 366(9494): p. 1367-74.
5. Gottlieb, A.B., et al., Certolizumab pegol for the treatment of chronic plaque psoriasis: Results through 48 weeks from 2 phase 3, multicenter, randomized, double-blinded, placebo-controlled studies (CIMPASI-1 and CIMPASI-2). J Am Acad Dermatol, 2018. 79(2): p. 302-314.e6.
6. Kavanaugh, A., et al., Safety and Efficacy of Intravenous Golimumab in Patients With Active Psoriatic Arthritis: Results Through Week Twenty-Four of the GO-VIBRANT Study. Arthritis Rheumatol, 2017. 69(11): p. 2151-2161.
7. Langley, R.G., et al., Secukinumab in plaque psoriasis--results of two phase 3 trials. N Engl J Med, 2014. 371(4): p. 326-38.
8. Griffiths, C.E., et al., Comparison of ixekizumab with etanercept or placebo in moderate-to-severe psoriasis (UNCOVER-2 and UNCOVER-3): results from two phase 3 randomised trials. Lancet, 2015. 386(9993): p. 541-51.
9. Lebwohl, M., et al., Phase 3 Studies Comparing Brodalumab with Ustekinumab in Psoriasis. N Engl J Med, 2015. 373(14): p. 1318-28.
10. Yan, K., et al., Efficacy and safety of vunakizumab in moderate-to-severe chronic plaque psoriasis: A randomized, double-blind, placebo-controlled phase 3 trial. J Am Acad Dermatol, 2025. 92(1): p. 92-99.
11. Warren, R.B., et al., Bimekizumab versus Adalimumab in Plaque Psoriasis. N Engl J Med, 2021. 385(2): p. 130-141.
12. Papp, K.A., et al., Efficacy and safety of ustekinumab, a human interleukin-12/23 monoclonal antibody, in patients with psoriasis: 52-week results from a randomised, double-blind, placebo-controlled trial (PHOENIX 2). Lancet, 2008. 371(9625): p. 1675-84.
13. Landells, I., et al., Ustekinumab in adolescent patients age 12 to 17 years with moderate-to-severe plaque psoriasis: results of the randomized phase 3 CADMUS study. J Am Acad Dermatol, 2015. 73(4): p. 594-603.
14. Blauvelt, A., et al., Efficacy and safety of guselkumab, an anti-interleukin-23 monoclonal antibody, compared with adalimumab for the continuous treatment of patients with moderate to severe psoriasis: Results from the phase III, double-blinded, placebo- and active comparator-controlled VOYAGE 1 trial. J Am Acad Dermatol, 2017. 76(3): p. 405-417.
15. Gordon, K.B., et al., Efficacy and safety of risankizumab in moderate-to-severe plaque psoriasis (UltIMMa-1 and UltIMMa-2): results from two double-blind, randomised, placebo-controlled and ustekinumab-controlled phase 3 trials. Lancet, 2018. 392(10148): p. 650-661.
16. Reich, K., et al., Tildrakizumab versus placebo or etanercept for chronic plaque psoriasis (reSURFACE 1 and reSURFACE 2): results from two randomised controlled, phase 3 trials. Lancet, 2017. 390(10091): p. 276-288.
17. Bachelez, H., et al., Trial of Spesolimab for Generalized Pustular Psoriasis. N Engl J Med, 2021. 385(26): p. 2431-2440.
